# Spontaneous parieto-occipital haematoma: lessons for the primary care clinician

**DOI:** 10.3399/bjgpopen20X101104

**Published:** 2020-06-24

**Authors:** Rajesh Pandey

**Affiliations:** 1 Priory Road Surgery, Hastings, UK

**Keywords:** Neurology, Family medicine, Hospital referrals, Primary health care, General practice

## Introduction

This case is intended to serve as a learning tool for all clinicians working in primary care. Headache is a commonly encountered problem in primary care and most cases do not mandate urgent transfer to secondary care. However, in this particular patient, there was focal neurology as well as a coital history of headache, both of which collectively mandated further investigation. She was diagnosed with a spontaneous parieto-occipital haemorrhage. On reflection, it is important to note that, despite this haemorrhage, the patient remained well enough to present to primary care and was still able to maintain a Glasgow Coma Scale score of 15/15. This case highlights two important areas of learning which could be useful to all healthcare professionals evaluating headaches. Firstly, any new focal neurology such as blurring of vision should prompt further investigation. Secondly, all healthcare professionals evaluating a sudden-onset headache should ask about whether the headache was coital or not, as this is very easy to ask and may prove to be very diagnostically useful.

## Case report

### Presenting complaint

A 44 year old woman presented to her GP on a morning walk-in clinic, complaining of an 18-hour history of severe headache which was spreading from the posterior to the anterior aspect of her head. She mentioned that this headache started the preceding evening and was made worse during sexual activity. She was also reporting some blurring of vision. Her observations measured in general practice were as follows: blood pressure of 120/80 mmHg; pulse of 82bpm and temperature of 36.3 degrees. On examination: her pupils were equal and reactive to light, GCS was 15/15, she also had a right-sided homonymous hemianopia.

### Contextual history

She had presented to the local emergency department 48 hours earlier with a similar posterior headache which started suddenly while she was having her evening meal. At this time, she had transient slurring of speech which spontaneously resolved. In the emergency department, the patient was found to have normal observations, with a mildly elevated white cell count of 13.2 (normal range: 4–11) with C-reactive protein <1, and erythrocyte sedimentation rate of 5.0 (normal range: 5.0–15.0). There were no focal neurological problems or red flag symptoms. Her renal profile and liver function tests were normal. Consequently, a working diagnosis of tension headache was made and the patient was discharged with advice to use over the counter analgesia.

She had a past medical history of depression, two previous miscarriages, and varicose veins. She was a smoker and took fluoxetine 40 mg once daily. Her history otherwise was normal. She had no relevant family or social history.

### Diagnostic work up

**Figure 1.**
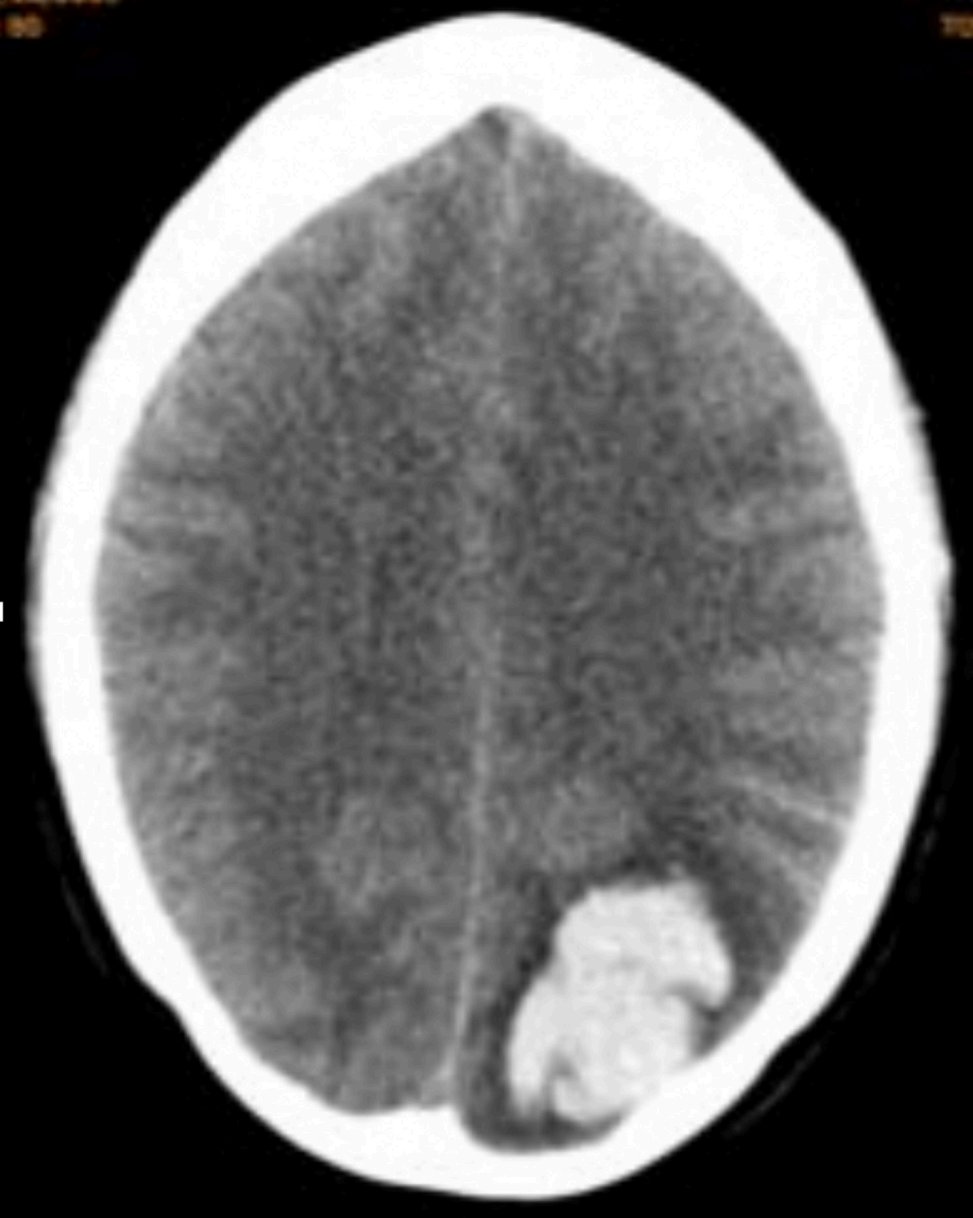
Head CT showing acute intraparenchymal haematoma within the left parieto-occipital lobe

During her appointment in general practice, it was clear that there was an intracranial pathology, although whether this was a haemorrhage or a solid mass was not known. As such, it was decided that this patient needed urgent transfer to secondary care in order to obtain a computed tomography (CT) scan of the brain. CT scan of her brain showed an ‘acute intraparenchymal haematoma within the left parieto-occipital lobes measuring 3.8 cm‘, with surrounding parenchymal oedema extending to the overlying cortex. This is shown in Figure 1.

### Treatment

The patient’s case was discussed with the on-call neurosurgical team, who advised that her case should be managed conservatively.

### Outcome and follow up

After the diagnosis of intraparenchymal haemorrhage was made, the patient underwent a brain CT angiogram which did not reveal any vascular cause of haemorrhage. Consequently, the patient was managed conservatively, with follow-up care organised by her neurovascular multidisciplinary team. She made an excellent recovery, with spontaneous resolution of her headache and improvement of her right sided hemianopia. As a result, the patient went back to work as copywriter. Eight weeks later, the patient underwent brain magnetic resonance imaging (MRI) and magnetic resonance angiography (MRA), which did not reveal any suspicious underlying abnormality. The patient also underwent a vasculitic screen which was normal. At the time of writing, the patient was 8 months post-diagnosis, she had made a complete recovery, and had a quality of life which was similar to that of a time before her diagnosis.

## Discussion

Spontaneous intracerebral haemorrhage (ICH) in young adults is a rare event. One case series from South Korea reviewed 39 consecutive cases of spontaneous ICH in patients under the age of 40 and found that 16 patients (41%) had no identifiable cause for developing ICH. Hence, these cases are labelled as cryptogenic ICH. The next most common causes were arteriovenous malformation (25.6%) and hypertension (23.1%).^1^

Unsurprisingly, the pathogenesis of cryptogenic ICH is poorly understood. There is, however, better data on prognostic features. Increasing age and size of haemorrhage are associated with poor prognosis. A case series by Daverat *et al* looked at prognostic outcome in 166 patients with spontaneous ICH. They reported that 6-month survival rate was 72% for patients aged <60 years, 55% for patients aged 60–69 years, and 43% for patients aged >69 years. It was suggested that mortality during the first 30 days was linked to the severity of the haemorrhage, and afterwards was linked to general functional status, which deteriorates with increasing age.^2^ Moreover, a retrospective Finnish study looking at long-term survival in 411 patients with primary ICH identified three independent predictors of death during long-term follow-up: old age, cardiac failure, and male sex.^3^ Reassuringly, none of these risk factors are present in the index patient.

It is important to recognise that clinicians working in primary care do not have the luxury of obtaining blood tests and performing lengthy clinical examinations; instead they need to ascertain the key features of the history and examination within minutes. This case highlights two key learning points designed to help all primary care clinicians evaluating headaches. Firstly, any new focal neurology such as blurring of vision should prompt further investigation. Secondly, all healthcare professionals evaluating a sudden-onset headache should ask about whether the headache was coital.
